# Flavonoids

**DOI:** 10.1016/j.advnut.2026.100611

**Published:** 2026-03-03

**Authors:** Alex Cheok, Ana Rodriguez-Mateos

**Affiliations:** Department of Nutritional Sciences, King’s College London, London, United Kingdom

**Keywords:** flavonoid, flavan-3-ol, cardiovascular, cardiometabolic, tea, berries, cocoa, nuts

## Flavonoids

**FIGURE 1:**
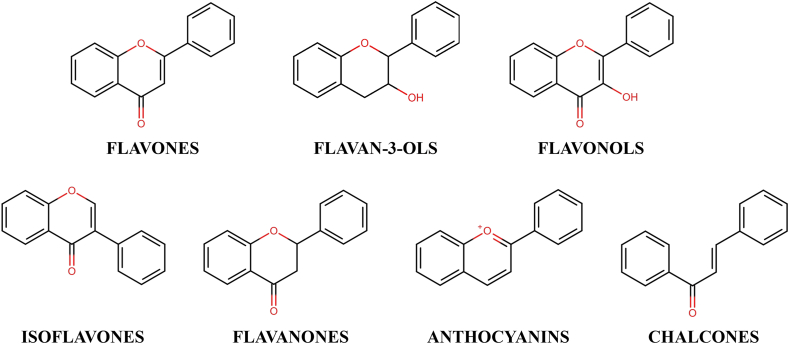
Basic skeleton structure of major flavonoid subclasses: flavones, flavan-3-ols, flavonols, isoflavones, flavanones, anthocyanins, and chalcones.

Flavonoids are by far the most abundant and diverse subclass of (poly)phenols in both plants and the human diet. They are plant secondary metabolites derived from the phenylpropanoid pathway, with over 10,000 structures identified to date [1]. Flavonoids are characterized by a 15-carbon backbone (C_6_-C_3_-C_6_) consisting of 2 benzene rings joined together by a three-carbon chain [2]. This, alongside different substitutions, makes up its subclasses (Figure 1). Notable groups of dietary flavonoids include the flavan-3-ols (e.g., epigallocatechin gallate in green tea), the flavonols (e.g., quercetin, kaempferol and myricetin from onions and kale), the isoflavones (e.g., daidzein and genistein from soybeans and legumes), the flavanones (e.g., naringenin in citrus fruits), the anthocyanins (e.g., cyanidin and delphinidin from berries and red cabbage), the flavones (e.g., luteolin and apigenin in parsley, chamomile and celery), and the chalcones (e.g., phloretin and butein in apples and hops). Most flavonoids in foods exist as glycosides, and their bioavailability is generally quite low [3]. This is because, unlike aglycones, glucosides are difficult to absorb and must first be hydrolyzed in the small intestine by enzymes such as lactase phlorizin hydrolase, either directly or first transported by membrane transporters like the sodium-dependent glucose transporter 1 and glucose transporter 2 prior to lactase phlorizin hydrolase hydrolysis [4]. Once inside the enterocytes and later in the liver, flavonoids undergo extensive biotransformation via phase 1 and 2 metabolism into flavonoid conjugates (glucuronides, sulfates, methylated derivatives). Unabsorbed flavonoids can reach the colon, where the gut microbiota plays a critical role in breaking them down into small phenolic compounds (e.g., hippuric acid and protocatechuic acid) that can be readily absorbed [3]. It is these conjugated metabolites and phenolic acids that circulate in plasma, which were thought to be bioactive and are responsible for many of the observed health effects attributed to flavonoid intake. Lastly, flavonoid metabolites are cleared from the system via urinary or biliary excretion (subsequently into feces) [5]. Flavonoids have a reasonable half-life *in vivo,* but can sometimes be further extended ≤11 h [6] because of multiple rounds of enterohepatic recycling, in which the metabolites are reabsorbed and recirculated [7].

### Deficiencies

Flavonoids are not essential nutrients, and no deficiency disorder is currently associated with inadequate flavonoid intake. However, there is evidence suggesting their consumption may be beneficial to health [8,9]. As flavonoids are among the predominant dietary bioactives in many fruits and vegetables, they may partly contribute to the association between low fruit and vegetable intake and higher mortality [10].

### Diet recommendations

A daily recommended intake (DRI) for flavonoids currently does not exist. Although flavonoids are not essential, there is moderate to strong evidence suggesting that flavonoids are important for the prevention of non-communicable diseases. One large-scale observational meta-analysis suggested that 500 mg/day intake of total flavonoids was associated with a 23%, 20%, and 27% lower relative risk of coronary artery disease, stroke, and cardiovascular disease (CVD), respectively [11]. This review is one of the most comprehensive in recent years, covering 39 prospective cohort studies involving over 1.5 million individuals. This effect on CVD risk was largely driven by the flavonoid subgroups—flavan-3-ols and anthocyanins; whereas flavonols and flavones were responsible for the improvement in coronary artery disease risk [11]. When looking at the diversity of flavonoid intake instead, a prospective cohort study of 124,805 UK Biobank individuals [12] showed that participants consuming the widest diversity of dietary flavonoids, flavonoid-rich foods and/or specific flavonoid subclasses experienced a 6% to 20% significantly lower risk of all-cause mortality and incidence of CVD, type 2 diabetes, cancer, respiratory disease and neurodegenerative disease. Both flavonoid quantity and diversity are independent predictors of these outcomes, suggesting that higher intake and wider diversity may be more beneficial for long-term health than either factor alone [12]. Beyond epidemiological evidence, there have been substantial clinical studies done on flavonoids, with multiple systematic reviews and meta-analyses conducted for all subclasses, with the strongest evidence currently existing for flavan-3-ols. A meta-analysis of 156 randomized controlled trials (RCTs) suggests that 40 to 1560 mg/d of flavan-3-ols gave rise to significant improvements in cardiometabolic risk factors, including endothelial function, blood lipid profile, blood pressure, hemoglobin A1c, and insulin resistance [13]. Altogether, the mounting evidence on flavan-3-ols has prompted the first-ever dietary bioactive guideline developed by the US Academy of Nutrition and Dietetics [14], which recommends a daily consumption of 400–600 mg flavan-3-ols for optimal cardiometabolic health. This amount is easily achievable in a normal diet (e.g., 2 cups of tea, a small bowl of berries, and an apple). An increasing body of evidence also exists for the effects of anthocyanins on cardiometabolic outcomes. An umbrella review of 8 meta-analyses, which included 139 RCTs, demonstrated improvements in blood lipids, glucose metabolism, and endothelial function, suggesting 200 to 400 mg/day of anthocyanins for cardiometabolic health [15]. Clinical evidence for other subclasses is also promising, in particular for soy isoflavones [16], flavonols [17], and flavanones [18].

### Food sources

According to the USDA database of flavonoid content of selected foods [19], major dietary sources of flavonoids include teas (black, green, oolong), red wine, fruits (berries, citrus, apples), cocoa (dark chocolate and cocoa powder), vegetables (colorful and cruciferous vegetables), legumes (soybeans and soy products), nuts (almonds and hazelnuts), seeds (flax and chia seeds), herbs (parsley and thyme), and spices (cinnamon, turmeric, cloves). Flavonoid content in tea, cocoa, and some berries may exceed 200 mg/100 g fresh weight [19].

### Clinical uses

There are currently no official therapeutic uses of flavonoids in clinical medicine. In the 2024 European Society of Cardiology Guidelines for the management of peripheral arterial and aortic diseases, the Mediterranean diet (a healthy diet rich in legumes, dietary fiber, nuts, fruits, and vegetables, with a high flavonoid intake) is the top recommendation for cardiovascular disease prevention in patients with peripheral arterial and aortic diseases. This flavonoid-rich diet has been assigned a class I recommendation (highest level), combined with level A (highest grade) for evidence. The guidelines were developed by the European Society of Cardiology-selected task force based on a critical review and evaluation of published literature on diagnostic and therapeutic approaches, which includes an assessment of the risk-benefit ratio [20]. They have been endorsed by the European Association for Cardio-Thoracic Surgery, the European Reference Network on Rare Multisystemic Vascular Diseases, and the European Society of Vascular Medicine.

### Toxicity

Due to the lack of an established DRI, there are currently no tolerable upper levels for flavonoids, and toxicity is generally not a concern when consumed from foods in a regular diet. Safety data in humans are limited for very high intakes, especially when taken as supplements or extracts. A meta-analysis of 34 RCTs on green tea extracts found that liver-related adverse events, particularly with elevated liver enzymes, were reported in only 4 studies when intake of epigallocatechin gallate exceeded 800 mg total daily dose [21]. They suggest that these effects are rare and reversible. Although this is not strictly an intolerance or a toxicity issue, consuming flavonoids (such as tea or coffee) during meals may hamper iron absorption [22] due to their natural affinity to chelate non-heme iron [23]. This is particularly a concern for individuals following a plant-based diet, as their primary source is often non-heme iron from plants. This inhibitory behavior is dose-dependent, in which single-meal iron absorption can be reduced by 60% to 90% when taken in conjunction with beverages containing 100–400 mg total (poly)phenols [24]. Anthocyanins are natural color pigments derived from berries and grapes. They have become an FDA-approved food colorant designated as grape color extract or E163 (in the EU) that is widely used in the food industry. In 1982, the Joint FAO/WHO Expert Committee on Food Additives suggested an average daily intake of 2.5 mg/kg body weight per day for grape skin anthocyanins (equivalent to 150 mg anthocyanins for a 60-kg adult). Later in 2013, the European Food Safety Authority re-evaluated this additive and concluded that the toxicological data were inadequate at that time to establish an average daily intake for anthocyanins [25]. However, they suggested that safety concerns are unlikely if intake could be comparable to those typically expected from the diet, implying that dietary intake of anthocyanins in food is generally safe.

## Recent research

Flavonoid research has a long history, yet interest in the field has surged in recent years and continues to grow. The investigation of their health benefits has been a primary focus, as reflected in large-scale epidemiological studies [11,12] together with hundreds of RCTs [13] exploring their preventative and therapeutic potential across a wide range of disease endpoints. Apart from evidence on cardiometabolic outcomes, there have also been promising data on other aspects, such as cancer risks [26,27] and cognitive function [28]. Currently, most evidence centers on flavan-3-ol and anthocyanin intake and its efficacy in promoting cardiometabolic health. The body of evidence for flavan-3-ols is by far the most comprehensive among all groups of plant bioactives, leading to the first-ever dietary recommendation (see Diet recommendations), bringing us one step closer to establishing a DRI for flavonoids. In the last decade, there has been an emerging interest in the bidirectional relationship between flavonoids (or polyphenols in general) and gut microbiota [29,30]. Gut microbes metabolize flavonoids to boost bioavailability and produce bioactive metabolites; flavonoids, in turn, influence gut environment and ultimately microbiota composition. This has led to the concept of the gut–brain axis, in which these circulating metabolites cross the blood–brain barrier and exert neurocognitive effects such as improving cognitive function and memory [31]. Despite considerable efforts [3,5], the true bioavailability of flavonoids remains partially elucidated due to poor absorption and extensive metabolism of flavonoids, so it is still unclear how much actually reaches target tissues. Furthermore, understanding their *in vivo* mechanisms of action remains a challenge because of complex interactions between flavonoids and the gut microbiota [7]. More research is warranted in these areas. This is an invited article to provide an update to an existing article [32].

## Author contributions

The authors’ responsibilities were as follows – AC and ARM were responsible for the manuscript conception, drafting, and final content; and all authors: read and approved the final manuscript.

## Funding

The authors reported no funding received for this study.

## Conflict of interest

The authors report no conflicts of interest.
